# Supplementary figures and images for: Shallow Whole-Genome Sequencing of Cell-Free DNA (cfDNA) Detects Epithelial Ovarian Cancer and Predicts Patient Prognosis
Source: Cancers (Basel). 2023 Jan 15;15(2):530. doi: 10.3390/cancers15020530 (PMC9857189; doi:10.3390/cancers15020530)

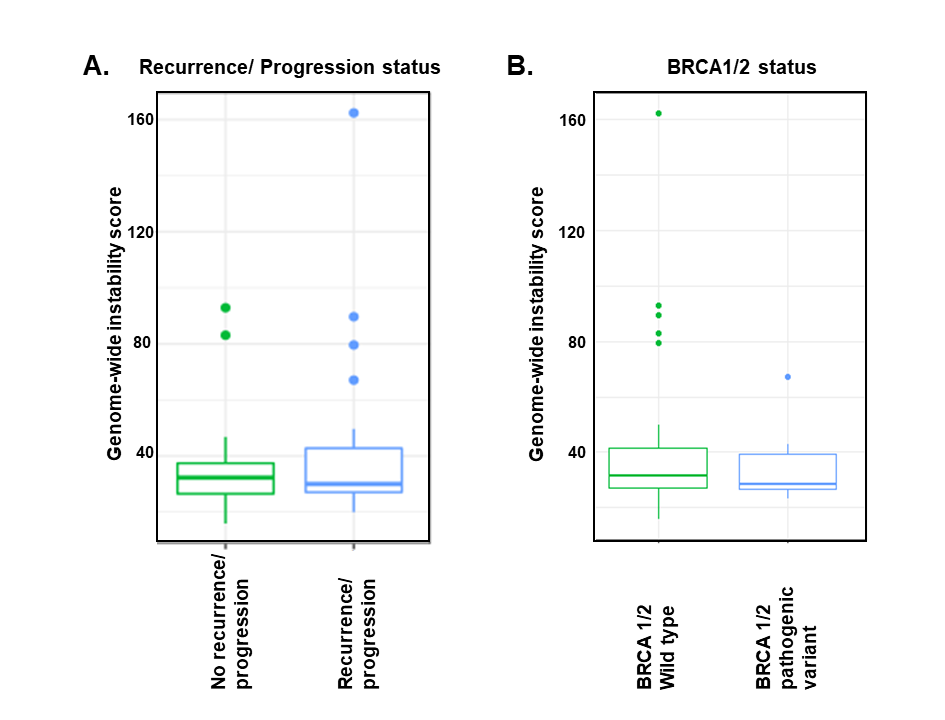

Supplement: Supplementary file 1 [file cancers-15-00530-s001.zip › FigureS1.tif]

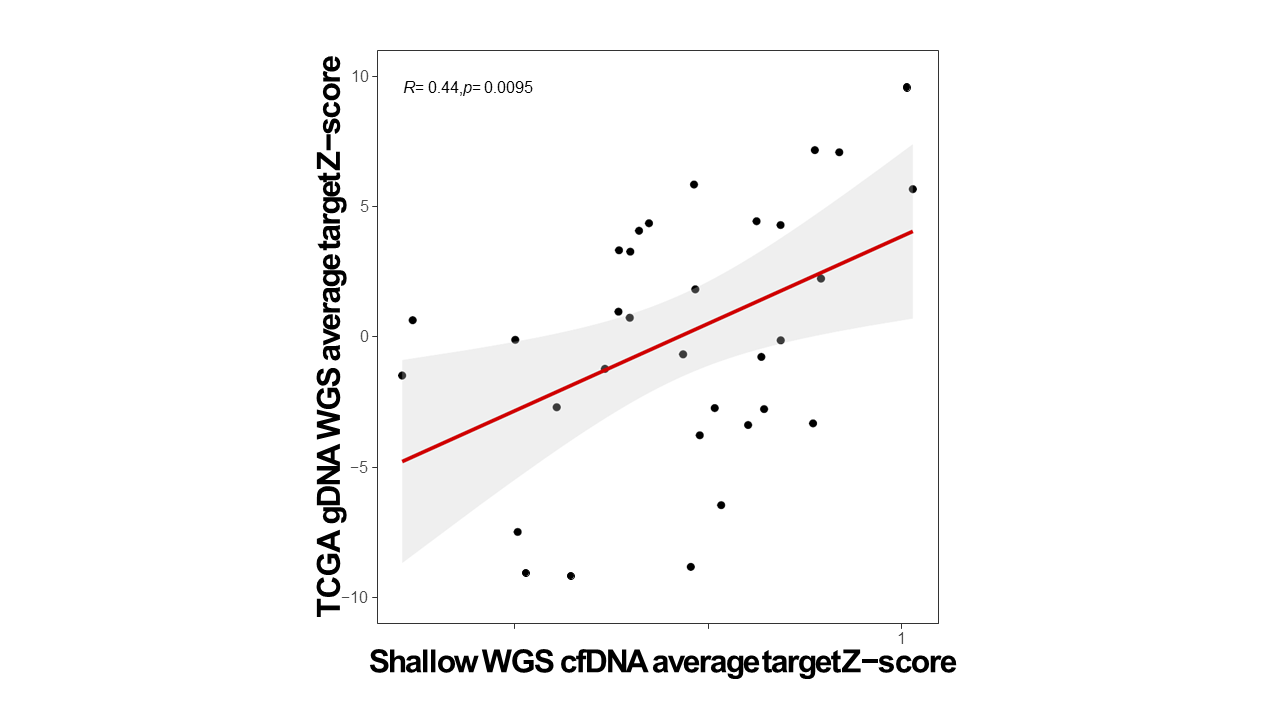

Supplement: Supplementary file 1 [file cancers-15-00530-s001.zip › FigureS2.tif]

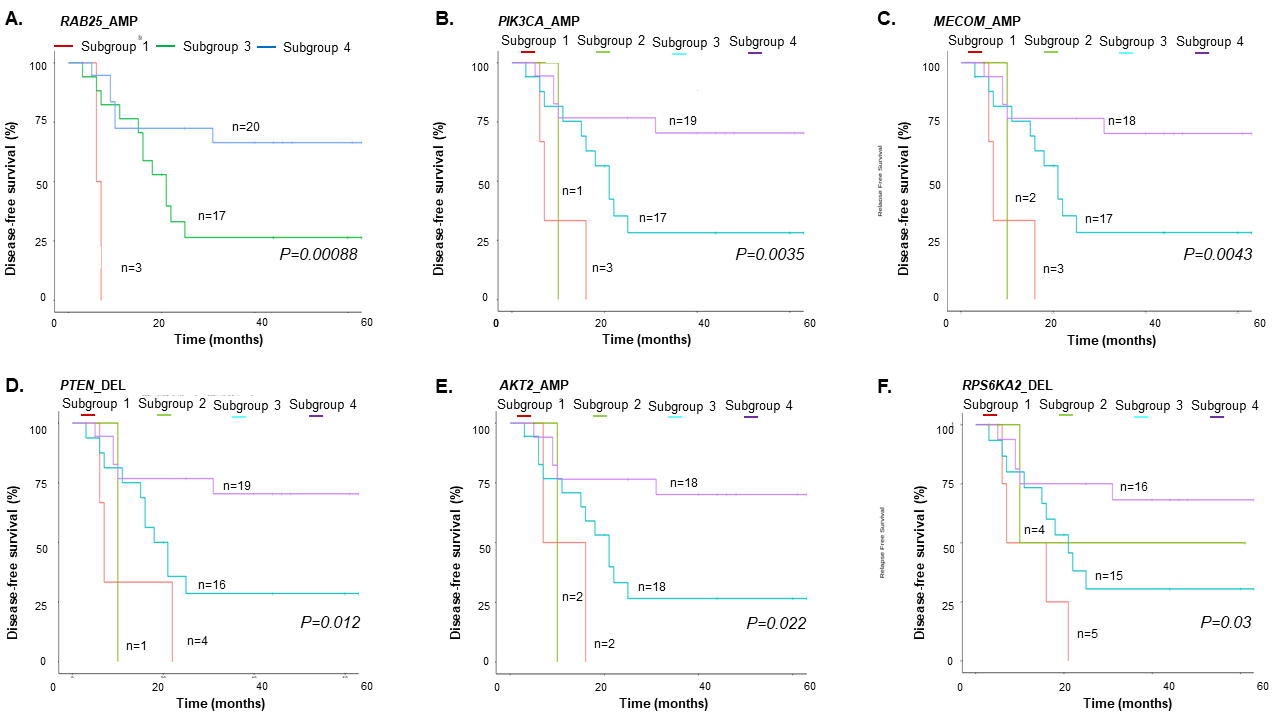

Supplement: Supplementary file 1 [file cancers-15-00530-s001.zip › FigureS3.tif]
